# Enhancement of blood-tumor barrier permeability by Sar-[D-Phe8]des-Arg9BK, a metabolically resistant bradykinin B1 agonist, in a rat C6 glioma model

**DOI:** 10.1186/1471-2202-5-38

**Published:** 2004-09-30

**Authors:** Ronie Cleverson Cardoso, Bruno Lobão-Soares, Marino Muxfeldt Bianchin, Carlos Gilberto Carlotti, Roger Walz, Márcio Alvarez-Silva, Andréa Gonçalves Trentin, Mauro Nicolau

**Affiliations:** 1 Departamento de Fisiologia, Universidade Federal de Santa Catarina, Brazil; 2 Departamento de Biologia Celular, Embriologia e Genética, Universidade Federal de Santa Catarina, Brazil; 3 Departamento de Neurologia, Psiquiatria e Psicologia Médica, Faculdade de Medicina de Ribeirão Preto, Universidade de São Paulo, São Paulo, Brazil; 4 Departamento de Cirurgia, Faculdade de Medicina de Ribeirão Preto, Universidade de São Paulo, São Paulo, Brazil; 5 Centro de Cirurgia de Epilepsia, Hospital Governador Celso Ramos, Florianópolis, Santa Catarina, Brazil; 6 Centro de Ciências da Saúde, Faculdade de Medicina da Universidade do Vale do Itajaí, UNIVALI, Itajaí, Santa Catarina, Brazil

## Abstract

**Background:**

While it is well known that bradykinin B2 agonists increase plasma protein extravasation (PPE) in brain tumors, the bradykinin B1 agonists tested thus far are unable to produce this effect. Here we examine the effect of the selective B1 agonist bradykinin (BK) Sar-[D-Phe8]des-Arg9BK (SAR), a compound resistant to enzymatic degradation with prolonged activity on PPE in the blood circulation in the C6 rat glioma model.

**Results:**

SAR administration significantly enhanced PPE in C6 rat brain glioma compared to saline or BK (p < 0.01). Pre-administration of the bradykinin B1 antagonist [Leu8]-des-Arg (100 nmol/Kg) blocked the SAR-induced PPE in the tumor area.

**Conclusions:**

Our data suggest that the B1 receptor modulates PPE in the blood tumor barrier of C6 glioma. A possible role for the use of SAR in the chemotherapy of gliomas deserves further study.

## Background

Bradykinin (BK) is a vasoactive peptide released from its high molecular weight precursors, the kininogens, through the action of serine proteases, the kallikreins, playing a crucial role in pathologic processes like inflammation, infectious diseases, and cancer [1,2]. The brain and spinal cord contain all of the components necessary for kinin formation and action. In addition, central nervous system traumas lead to kinin formation [3]. BK type-1 (B1) receptors are mostly expressed in pathological or stressful situations related to tissue damage [1,2] including tumors [2]. The BK type-2 (B2) receptor is defined as a constitutive one, being normally encountered in many tissues, including the brain [2,4]. It is well known that the blood-brain barrier (BBB) in brain tumor regions (also called blood tumor barrier, or BTB) shows different plasma protein extravasation (PPE) characteristics when compared with the BBB of normal brain tissue and this effect is particularly related to the increase of B2 receptors [5-7]. In animal models, B2 receptor agonists like Cereport^® ^(RMP-7) have been used by the intracarotid route to enhance the delivery of chemotherapeutic agents to the brain tumor area [7-9]. B1 receptor agonists with low metabolic resistance (MR) have already been unsuccessfully tested on PPE extravasation through the BTB in brain tumors [8,9]. The B1 receptor agonist, Sar-[D-Phe8]des-Arg9BK (SAR), shows some resistance to enzymatic degradation that is due to the addition of amino acids to the amino-terminal portion of the molecule [10]. Thus, it is possible that SAR could be successful in promoting PPE through the BTB, having potential uses in enhancing the delivery of chemotherapeutic agents to brain tumors. In order to test this hypothesis, we investigated the effect of SAR on PPE using the rat C6 glioma model [11].

## Results

**Figure 1:**
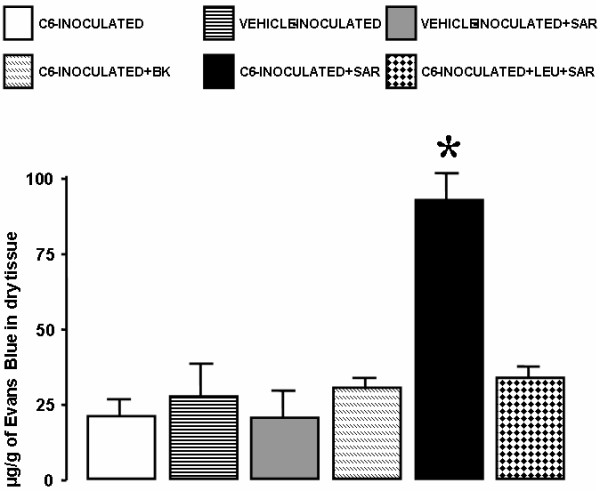
Protein plasma extravasation (PPE) in the right (inoculated) hemisphere of 6 different groups of animals. There was a significant increase in PPE when the 17-day-C6-injected animals were concomitantly injected with SAR 100 nm/Kg (C6 + SAR) compared to the control group (C6-inoculated). Vehicle-inoculated and vehicle-inoculated + SAR (100 nm/Kg) animals did not differ from each other or from the control. Bradykinin (100 nm/Kg) injected through the femoral vein did not enhance BTB permeability (C6 inoculated + BK). However, the specific bradykinin B1 antagonist, LEU (100 nm/Kg), improved SAR-induced PPE in the tumor mass (C6-inoculated + LEU + SAR). *p < 0.01 compared to PPE in the C6-inoculated, vehicle-inoculated, vehicle-inoculated + SAR, and C6 inoculated + BK groups and in the C6-inoculated+LEU+SAR group.

**Figure 2:**
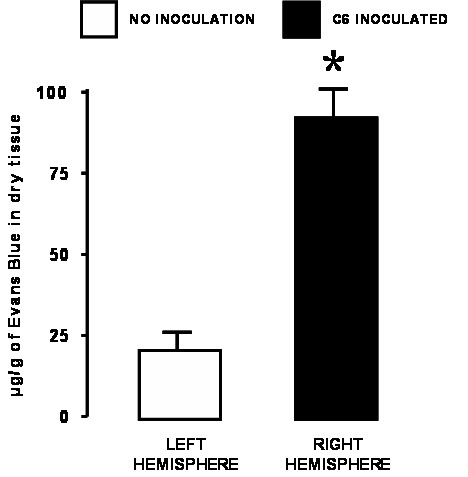
Protein plasma extravasation (PPE) in the right (C6-inoculated) and left (non-tumoral) hemisphere of rats. The analysis was performed 17 days after the inoculations. PPE was significantly increased in the tumor hemisphere compared to the non-tumoral one. *p < 0.001 compared to PPE in the control (left) hemispheres.

Eighty percent of C6-inoculated animals developed brain tumors after a period of 30 days (data not shown). We determined PPE in experimental groups of animals treated with bradykinin (BK), the BK receptor B1 agonist (SAR) or the BK receptor B1 antagonist (LEU) (Fig. 1). PPE in the tumor area of C6-inoculated animals was 22.8 ± 6.4 μg EB/g dry tissue, a value that did not differ from those observed in tumor-free animals inoculated with vehicle only (26.3 ± 16.7 μg EB/g dry tissue), or inoculated with cell vehicle plus SAR (23.2 ± 9.8 μg EB/g dry tissue). Injection of BK into C6-inoculated animals also did not change the tumor PPE (30.6 ± 10.9 μg EB/g dry tissue). It is important to note that in all groups the left (non-inoculated) hemispheres presented PPE values similar to those of the right (inoculated) hemisphere in the control animals. However, SAR significantly increased PPE in the tumor area of C6-inoculated animals (91.9 μg EB/g dry tissue ± 7.9) (*p *< 0.01). This effect was abolished, with a return to basal levels, by pre-treatment of the animals with the BK receptor B1 antagonist LEU (35.8 ± 8.6 μg EB/g dry tissue). The effect of SAR in increasing PPE in C6-inoculated right hemispheres (91.9 μg EB/g dry tissue ± 7.9) was also observed (t = 24.9, *p *< 0.001) when compared to the contralateral non-tumoral hemisphere (21.7 μg EB/g dry tissue ± 3.0, *n *= 6) (Fig. 2).

## Discussion

We demonstrate here that the selective B1 receptor agonist SAR [16] enhances PPE in C6 glioma without affecting the normal brain parenchyma. It has been previously shown that SAR at 100 nmol/Kg also does not alter the systemic blood pressure of rats [17]. The effect of SAR was blocked by pre-treatment with the selective B1 receptor antagonist LEU, suggesting that PPE can be modulated by B1 receptors in the glioma model of C6-inoculated rats.

It was recently described that gliomas may express both B1 and B2 BK receptors, with B2 being located closer to the periphery of the tumor cells while B1 was observed throughout the cell [18]. In view of their effects on vascular dilatation and blood flow, bradykinin receptor agonists have been tested in the chemotherapy of brain tumors [18,19], whereas bradykinin itself was less used for this purpose [6,7] due to its lower efficacy and shorter blood half-life. In contrast to other authors [6,9] we did not observe an effect of BK by itself in enhancing BBB or BTB permeability. Fast BK degradation could account for this difference since we used a femoral vein, whereas other authors used more proximal accesses like the carotid artery [6,9]. The B2 receptor agonist RMP-7 (Cereport^®^) has been successfully employed to enhance BTB permeability to chemotherapeutic agents [7,20], but in the animal models already tested only local or regional routes like the carotid artery were used [20,21].

In general, B1 receptors are non-constitutive [3] and restricted to pathologic processes including tumors or degeneration [2]. Systemically administered metabolically resistant B1 receptor agonists may be useful to increase chemotherapy levels at the target site, with higher specificity and lower toxicity than B2 receptor agonists. Thus, we believe that SAR injected together with chemotherapeutic agents may be useful to increase drug delivery specifically to the brain tumor with less toxicity to non-affected areas. Additionally, because EB circulates bound to albumin, it is reasonable to think that SAR could increase the delivery of several chemotherapeutic agents of molecular weight similar to, or lower than that of albumin [22,23]. Although the Evans Blue method is less sensitive than other labeling methods like quantitative autoradiography, for example, we used it because it was the only method available to us at the time of this experiment. However, the Evans-Blue method is of low cost, can be easily reproduced by other laboratories, and was sensitive enough to detect differences provoked by SAR in these experiments.

BK or BK agonists have been previously used to increase BTB permeability but, due to their faster enzymatic degradation [6,8], they were injected into the carotid artery. However, our findings suggest, that SAR administered intravenously remains stable and thus could be an attractive agent for the treatment of brain or even other tumors [24,25] that express BK B1 receptors.

## Conclusions

Our data suggest that the B1 receptor modulates PPE in the blood tumor barrier of C6 glioma. A possible role for the systemic use of SAR in the chemotherapy of gliomas or other CNS neoplasms deserves further study.

## Methods

### C6 glioma cell culture

The C6 rat glioma cell line was cultured as previously described [12]. Briefly, cells were grown in Dulbecco's modified Eagle medium (DMEM, Sigma, Saint Louis, MO, USA) supplemented with 5% fetal calf serum (FCS, Cultilab, Brazil), 100 mg/ ml streptomycin and 100 U/ ml penicillin (all from Sigma) under standard culture conditions. Cells were harvested with 0.125% trypsin/0.78 mM EDTA when they reached confluence.

### Animals and tumor inoculation

Male Wistar rats (aged 3 months and weighing 250–300 g upon arrival from the UFSC Central Animal House) were used. The rats were maintained in a temperature- and light-controlled vivarium (22°C; 12:12-h light:dark cycle; lights on at 07:00 h), with food and water available *ad libitum*. Animals were treated according to the Guidelines for the Use of Animals of Universidade Federal de Santa Catarina. Before the procedures, the rats were anesthetized with sodium pentobarbital (50 mg/Kg body weight, i.p.) and then injected with 1 × 10^6 ^C6 cells prepared in 5 ml DMEM/FCS (vehicle) into the right cerebral hemisphere using the following coordinates: 4 mm lateral from right bregma and 4.5 mm deep from the dural surface, as previously described [14].

### Pharmacological treatment and protein plasma extravasation (PPE) studies

Seventeen days after C6 inoculation, BBB or BTB extravasations were assessed by the Evans blue (EB, Sigma) method [14]. EB (20 mg/Kg; 25 mg/ml in 0.9% NaCl) was administered intravenously via a femoral vein with saline (*n *= 10, C6-inoculated; *n *= 6, control) or in combination with 100 nmol/Kg bradykinin (RBI, USA) (*n *= 6, C6-inoculated) or with 100 nmol/kg of selective bradykynin B1 agonist Sar-[D-Phe8]des-Arg9BK (*n *= 9, C6 inoculated; *n *= 6, control) (SAR, a gift from Dr Domenico Regoli, Université de Sherbrooke, Sherbrooke, Québec, Canada). An additional group of C6-inoculated animals (*n *= 6) were treated intravenously with SAR (100 nm/Kg) 5 min after pre-treatment with 100 nmol/Kg of the B1 receptor antagonist [Leu8]-des-Arg9BK (LEU, Sigma). Previous pilot studies conduced in our laboratory (data not presented) showed that SAR had a maximum effect at the equivalent dose of 100 nmol/Kg. These assays also showed that LEU at the dose used was effective in blocking the effect of SAR effect on isolated brain parenchyma and dura-mater. Fifteen minutes after the treatments, rats were perfused with 4 ml/Kg 0.9% NaCl for 3 min [14] and the brains were removed. Two cubic centimeters of tissue were removed around the inoculation site (right hemisphere) and from the contralateral homologous region (left hemisphere). EB was extracted with formamide (4 ml/g of wet weight tissue at room temperature for 24 h) and quantified at 620 nm with a Titerk Multiscan Microplate reader by comparison with a standard curve of EB (0.5 to 25 μg/ml of formamide). Extravasation was expressed as microgram of EB per gram of dry tissue.

### Statistical analysis

Differences between treatments were evaluated by one-way ANOVA followed by the Bonferroni post-hoc test (plasma extravasations studies) or by the Student t-test for paired samples (for comparisons between right and left cerebral hemispheres). Values were considered statistically significant when *p *< 0.05.

## List of abbreviations used

**BBB-**Blood brain barrier

**BK-**Bradykinin

**BTB-**Blood tumor barrier

**B1-**Bradykinin receptor B1

**B2-**Bradykinin receptor B2

**EB-**Evans Blue

**LEU-**[Leu8]-des-Arg9BK

**MR-**Metabolic resistance

**PPE-**Plasma protein extravasation

**SAR-**Sar-[D-Phe8]des-Arg9BK metabolically resistant bradykinin B1 agonist

## Authors' contributions

BLS and RCC contributed equally to this work. BLS and RCC carried out the surgical procedures and cell culture. MMB, RW, and CGCJr participated in the preparation of the manuscript. MAS carried out the absorbance assays. AGT and MN conceived the study and participated in its design and coordination. All authors participated in the analysis of the data and discussion of the results. All authors read and approved the final manuscript.
